# Results of a Randomized Clinical Study of Gemcitabine Plus Nab-Paclitaxel Versus Gemcitabine Plus S-1 as Neoadjuvant Chemotherapy for Resectable and Borderline Resectable Pancreatic Ductal Adenocarcinoma (RCT, CSGO-HBP-015)

**DOI:** 10.1245/s10434-024-15199-8

**Published:** 2024-03-28

**Authors:** Daisaku Yamada, Shogo Kobayashi, Hidenori Takahashi, Yoshifumi Iwagami, Hirofumi Akita, Kei Asukai, Junzo Shimizu, Terumasa Yamada, Masahiro Tanemura, Shigekazu Yokoyama, Masanori Tsujie, Tadafumi Asaoka, Yutaka Takeda, Osakuni Morimoto, Akira Tomokuni, Yuichiro Doki, Hidetoshi Eguchi

**Affiliations:** 1 Department of Gastroenterological Surgery, Graduate School of Medicine, Osaka University, Suita, Japan; 2 Department of Gastroenterological Surgery, Osaka International Cancer Institute, Osaka, Japan; 3 Department of Gastroenterological Surgery, Toyonaka Municipal Hospital, Toyonaka, Japan; 4 Department of Gastroenterological Surgery, Higashiosaka City Medical Center, Higashiosaka, Japan; 5 Department of Gastroenterological Surgery, Rinku General Medical Center, Izumisano, Japan; 6 Department of Gastroenterological Surgery, Hyogo Prefectural Nishinomiya Hospital, Nishinomiya, Japan; 7 Department of Gastroenterological Surgery, Osaka Rosai Hospital, Sakai, Japan; 8 Department of Gastroenterological Surgery, Osaka Police Hospital, Osaka, Japan; 9 Department of Gastroenterological Surgery, Kansai Rosai Hospital, Amagasaki, Japan; 10 Department of Surgery, Japan Community Health Care Organization Osaka Hospital, Osaka, Japan; 11 Department of Gastroenterological Surgery, Osaka General Medical Center, Osaka, Japan

**Keywords:** NAC, R/BR-PDAC, GEM+nPTX, GEM+S-1, R-PDAC

## Abstract

**Background:**

The optimal neoadjuvant chemotherapy (NAC) regimen for patients with localized pancreatic ductal adenocarcinoma (PDAC) remains uncertain. This trial aimed to evaluate the efficacy and safety of two neoadjuvant chemotherapy (NAC) regimens, gemcitabine plus nab-paclitaxel (GA) and gemcitabine plus S-1 (GS), in patients with resectable/borderline-resectable (R/BR) PDAC.

**Patients and Methods:**

Treatment-naïve patients with R/BR-PDAC were enrolled and randomly allocated. They received two cycles (2 months) of each standard protocol, followed by radical surgery for those without tumor progression in general hospitals belonging to our intergroup. The primary endpoint was to determine the superior regimen on the basis of achieving a 10% increase in the rate of patients with progression-free survival (PFS) at 2 years from allocation.

**Results:**

A total of 100 patients were enrolled, with 94 patients randomly assigned to the GS arm (*N* = 46) or GA arm (*N* = 48). The 2-year PFS rates did not show the stipulated difference [GA, 31% (24–38%)/GS, 26% (18–33%)], but the Kaplan–Myer analysis showed significance (median PFS, GA/GS 14 months/9 months, *P* = 0.048; HR 0.71). Secondary endpoint comparisons yielded the following results (GA/GS arm, *P*-value): rates of severe adverse events during NAC, 73%/78%, *P* = 0.55; completion rates of the stipulated NAC, 92%/83%, *P* = 0.71; resection rates, 85%/72%, *P* = 0.10; average tumor marker (CA19-9) reduction rates, −50%/−21%, *P* = 0.01; average numbers of lymph node metastasis, 1.7/3.2, *P* = 0.04; and median overall survival times, 42/22 months, *P* = 0.26.

**Conclusions:**

This study found that GA and GS are viable neoadjuvant treatment regimens in R/BR-PDAC. Although the GA group exhibited a favorable PFS outcome, the primary endpoint was not achieved.

**Supplementary Information:**

The online version contains supplementary material available at 10.1245/s10434-024-15199-8.

Pancreatic ductal adenocarcinoma (PDAC) is a lethal disease because the tumor cells have a tendency to spread to the surrounding areas and/or distant organs, allowing PDAC to become a systemic disease from an early stage.^1^ Imaging of localized PDAC, including resectable/borderline resectable PDAC (R/BR-PDAC), may not truly reflect the extent of localization. Merely resecting the tumor does not ensure a cure,^2^ and it appears that there may be the presence of tumor seeds concealed within the patient’s body, not detectable by imaging even in the localized stage. Thus, multimodal strategies, including surgery plus pre/postoperative therapies (i.e., neoadjuvant chemotherapy, NAC/adjuvant chemotherapy, AC), have been attempted to improve the surgical outcomes of patients with R/BR-PDAC.^2–11^

NAC is a standard treatment for BR-PDAC, supported by clinical evidence.^12–15^ Although the benefits of NAC for R-PDAC were debated,^7, 8, 13, 14, 16^ recent meta-analyses suggest it improves overall survival by increasing the number of patients with negative lymph node metastasis at surgery.^14, 16^ Moreover, several prospective studies have suggested the superiority of a preoperative treatment strategy over upfront surgery.^17-19^ Since the adopted regimens in those studies varied widely, the optimal NAC regimen for patients with R/BR-PDAC remains unclear, and clinical trials to explore the better regimen of NAC for R/BR-PDAC are now ongoing worldwide.

This phase II trial was designed to examine the efficacy and safety of two regimens, gemcitabine plus nab-paclitaxel (GA) and gemcitabine plus S-1 (GS), as NAC in patients with R/BR-PDAC, focusing on progression-free survival (PFS) as the primary endpoint. We planned to evaluate two treatment strategies that incorporated these NAC regimens prior to the standard treatment, which included upfront surgery followed by AC according to the prevailing protocol at that time. The rationale behind the GS regimen was based on phase II and subsequent phase III trials for R/BR-PDAC (mainly targeted R-PDAC) in which NAC-GS demonstrated clinical advantages over upfront surgery with acceptable feasibility in Japan (PREP-02).^17, 20^ The rationale behind the GA regimen was based on a phase III trial that showed a higher objective response rate for GA therapy than for GEM monotherapy against unresectable PDAC,^21^ and several studies demonstrated that the GA regimen was safely performed in patients with R/BR-PDAC.^SPS:refid::bib2222^ Given these findings, both NAC treatment regimens could be safely combined with the standard treatments. However, the comparison between these regimens as NAC treatment for patients with R/BR-PDAC has not been examined.

## Patients and Methods

The details of this study protocol have been previously described.^23^ All authors had access to the study data and reviewed and approved the final manuscript.

### Study Oversight

This trial (CSGO-HBP-015) was a multicenter, two-arm, open-label, randomized, exploratory trial with two treatment arms (GA/GS arm) allocated in a 1:1 ratio. Participants were stratified according to the institution and serum carbohydrate antigen 19-9 value (CA19-9, < 370 U/ml versus ≥ 370 U/ml) within 2 weeks of the eligibility screening. The trial was led by an intergroup, the Clinical Study Group of Osaka University, Hepato-Biliary Pancreatic Group (CSGO-HBP), in Japan. Eligible patients were recruited and treated in 11 of those hospitals and were centrally registered at a nonprofit organization, the Supporting Center for Clinical Research and Education (SCCRE), Osaka, Japan. Block randomization was conducted via a computer-generated random number list prepared by SCCRE, and the allocation sequence was concealed from the researchers.

All physicians involved in clinical trials in Japan underwent good clinical practice training and protocol training. Written informed consent was obtained before enrollment.

### Patients

Patients were over 20 years of age, with histologically confirmed treatment-naïve PDAC, an Eastern Cooperative Oncology Group performance status (PS) of 0 or 1, and localized tumor without distant metastasis. We intended to enroll patients with anatomically resectable PDAC and included patients with resectable PDAC according to our criteria of resectability at the time the trial was designed. Thus, not only R-PDAC, but also a part of BR-PDAC according to the present classification of the National Comprehensive Cancer Network (NCCN, NCCN guidelines version 2.2021) were included. The details of inclusion criteria were described previously.^23^ Both the patients with BR-portal vein (PV) and those with the tumor abutting the inferior vena cava (IVC) were allowed to participate. Concerning BR-artery (A)-PDAC, only when the tumor was located in the pancreatic body or tail were the patients eligible even if the tumor was in contact with arterial abutments, including the hepatic artery and/or celiac artery. Therefore, after completing the enrollment of the final patient, we collected data on vascular invasion for all cases to reclassify the cases according to the NCCN criteria for R/BR classification. Specifically, 18 cases of BR-PV and 2 cases of BR-A were included, with no patients exhibiting involvement of the abutted IVC.

### Treatment

#### Neoadjuvant Chemotherapy (NAC)

Patients allocated to the GA arm received intravenous GEM and subsequent nab-paclitaxel (nab-PTX) at doses of 1000 mg/m^2^ and 125 mg/m^2^, respectively, according to their body surface area (BSA) on days 1, 8, and 15 of a 28-day cycle.

Patients allocated to the GS arm received intravenous gemcitabine at a dose of 1000 mg/m^2^ on days 1 and 8 plus S-1 orally at a dose according to their BSA (< 1.25 m^2^, 40 mg; BSA 1.25–1.5 m^2^, 50 mg; BSA > 1.50 m^2^, 60 mg) twice daily on days 1–14 of a 21-day cycle.

These neoadjuvant treatments were repeated for two cycles unless there was unacceptable toxicity. Restaging by computed tomography (CT) was required before surgery. In cases of unexpected tumor progression (unresectable tumor extension or distant metastasis), patients received palliative treatment, including chemotherapy and/or radiotherapy, as off-protocol care.

### Surgery

Patients who received NAC treatment underwent surgery within 4–7 weeks after the last administration of chemotherapy if tumor progression was not detected. In our institutes, the surgical margin of the pancreas was examined intraoperatively using rapid pathological examination. In cases where a positive margin was detected, additional resection was performed until negative confirmation was obtained through additional rapid pathological examination. In cases of unexpected intraoperative findings regarding unresectability, including distant metastasis or inseparable tumor extension into major arteries, patients did not undergo pancreatectomy but underwent a suitable bypass procedure if necessary.

### Adjuvant Chemotherapy (AC) and Follow-Up

To evaluate the NAC treatment in addition to the standard treatment, this study did not stipulate AC. The standard treatment according to the national guidelines at that time consisted of curative resection and AC (primarily S-1, otherwise GEM), and AC was strongly recommended for cases with R0 or R1 resection. In practice, the majority of cases in this study received AC with S-1.

Follow-up observations were performed as described previously.^24^ To investigate recurrence, both serum level of tumor markers (i.e., CA19-9) and radiological imaging (i.e., CT) were examined every 3–4 months. The date of recurrence was defined as the date on which the investigator detected recurrence on an image or in a biopsy specimen.

### Endpoints and Assessments

The primary endpoint of this study was PFS at 2 years. On the basis of the published literature, which argued for the efficacy of AC at the time of the study design, we assumed that the superior NAC treatment would increase the 2-year PFS of patients by more than 10%, compared with the inferior NAC treatment. This assumption was based on the results of previous clinical studies on AC, such as CONKO-001 and JASPAC-01, which reported a 14–20% increase in PFS with AC.^2, 3^ We anticipated that the new hopeful perioperative treatment would yield a similar level of improvement. For the two arms with superior PFS at 2-year increase of 10% compared with inferior PFS to have at least 80%, 85%, and 90% probabilities of selecting the better arm, we need sample sizes (*N*) of 33, 50, and 76 patients per arm, respectively. The planned total sample size is at least 100 with 85% power, with a superior PFS at 2-year increase of 10% compared with the inferior arm. PFS was calculated from the day of randomization to the day of death from any cause or to the day of tumor progression, and was censored on the last day that the patient was documented to be alive without tumor progression. To calculate the rate of patients with progression-free status at 2 years, additional information on tumor progression at the 24-month mark after allocation was collected for four patients whose observation time did not reach 24 months at the last follow-up date. Tumor progression was defined as the appearance of a new lesion on the image or according to the surgeon’s findings during surgery. If the growth of the primary lesion expanding to an unresectable lesion was detected before surgery, the tumor was assumed to have progressed. The detection of any recurrence site was considered tumor progression after resection. We did not define recurrence solely on the basis of an increase in tumor markers. Information on tumor progression types was collected to evaluate each rate.

The secondary endpoints were resection rate, relative dose intensity (RDI), responses for both NAC arms, recurrence type, overall survival (OS), and adverse events. The resection rate was defined as the proportion of resection cases after either NAC treatment. OS was calculated from the day of randomization to the day of death from any cause and was censored on the last day that the patient was documented as alive. As an evaluation of radiological responses, reduction rate of tumor diameter in CT images was evaluated at the timepoint after NAC performance. Change in the serum value of tumor markers was estimated at the same timepoint as response to NAC.^25^ Pathological response was diagnosed by specialized pathologists at each institution according to the Evans classification.

### Statistical Analysis

Both PFS and OS were based on the intent-to-treat (ITT) population, which included all eligible patients allocated in the study. The primary endpoint of PFS at 2 years after allocation was assessed by using a timepoint evaluation. Kaplan–Meier analysis was used to construct survival curves and to evaluate differences in both PFS and OS (Wilcoxon test). The associated hazard ratio (HR) and two-sided 95% confidential index (CI) were provided using the stratified Cox proportional hazards model. For the comparison of the other outcomes, the chi-squared test and Fisher’s exact test were used. All analyses were conducted with the JMP 14 software program (SAS Institute, Cary, NC, USA).

### Trial Registration

UMIN Clinical Trials Registry UMIN000021484 (https://center6.umin.ac.jp/cgi-open-bin/ctr_e/ctr_view.cgi?recptno=R000024781). This trial began in April 2016.

### Ethics Approval and Consent to Participate

This study protocol and informed consent forms were approved by the ethics committee of each participating institution. All physicians involved in clinical trials in Japan underwent good clinical practice training and protocol training. Written informed consent was obtained before enrollment. This study was performed in accordance with the Declaration of Helsinki.

## Results

All analyses were conducted using data collected at the data cutoff (14 December 2022). The median follow-up time was 22 (CI 19–27) months.

### Patients

**Fig. 1:**
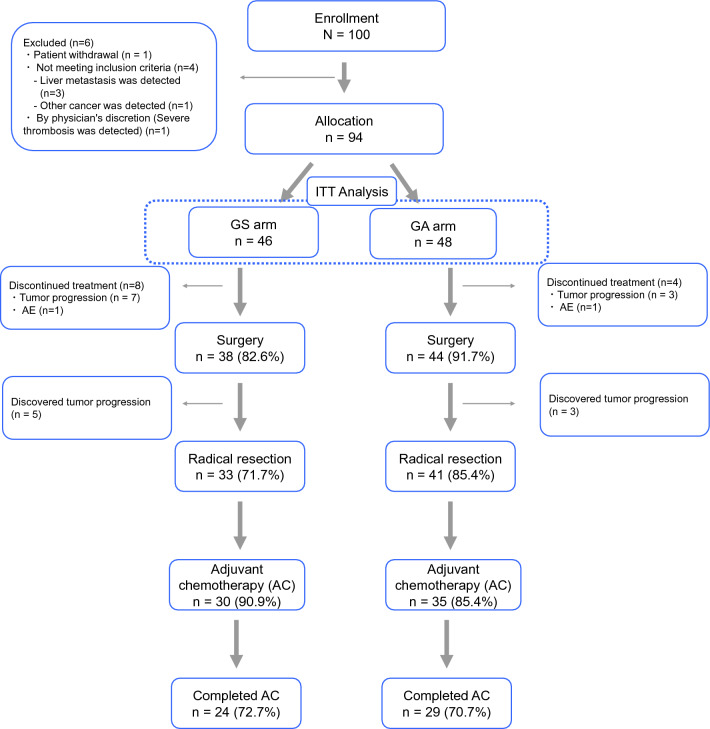
CONSORT diagram; flow diagram results between treatment arms are depicted. *GS arm* gemcitabine + S-1 regimen arm, *GA arm* gemcitabine + nab-paclitaxel arm, *ITT* intention to treat, *AE* adverse event

A total of 100 patients were enrolled between April 2016 and August 2021 in Japan, and one of those patients withdrew consent after enrollment. After excluding the 5 ineligible patients, 94 patients were allocated and randomly assigned to receive the GS arm (*N* = 46) or GA arm (*N* = 48) (Fig. 1).

Demographic and baseline characteristics were balanced between arms (Table 1). In the cohort of 94 patients, the median age was 69 (range 46–84) years, including 45 men (48%), and 94% of cases were categorized as PS 0. Biliary drainage was performed in 41% of patients before enrollment, and 31% of patients had diabetes mellites. The number of patients with R-PDAC was 74 (79%), and 20 patients with BR-PDAC were included. There was no difference between arms (R/BR; GA, 40/8; GS, 34/12; *P* = 0.265).

**Table 1:** Patients’ characteristics

	GS arm	GA arm
	*N* or mean ± SD	
Age	66 ± 1.3	68 ± 1.3
Sex (male/female)	21/25	24/24
Biliary drainage (−/+)	25/21	30/18
PS (0/1)	43/3	45/3
DM (−/+)	31/15	34/14
Tumor diameter (mm)	25.5 ± 1.2	23.3 ± 1.2
Tumor location (Ph/Pb/Pt/other)	25/14/5/2	32/9/6/1
CA19-9 (U/ml)	1241.2 ± 412.9	992.7 ± 404.2
CA19-9 < 370/370 ≦	14/32	14/34
CEA (ng/ml)	18.8 ± 9.2	4.3 ± 9.1
DUPAN-2 (U/ml)	1151.1 ± 319.1	403.6 ± 335.5
UICC cT (1/2/3/4)*	14/28/3/1	19/27/2/0
UICC cN (0/1+2)*	34/12 (12+0)	41/7 (6+1)
UICC cStage (IA/IB/IIA/IIB/III/IV)*	13/19/2/11/1/0	19/21/1/6/1/0
NCCN R/BR	34/12	40/8

^*^TNM classification was according to the 8th UICC classification.

*GS arm* gemcitabine + S-1 regimen arm, *GA arm* gemcitabine + nab-paclitaxel arm, *PS* performance status, *DM* diabetes mellites, *Ph* pancreas head, *Pb* pancreas body, *Pt* pancreas tail, *NCCN* National Comprehensive Cancer Network, *R* resectable, *BR* borderline resectable

### Treatment

#### Neoadjuvant Chemotherapy (NAC)

The average RDI that patients in the GA arm received was 85% (nab-PTX) and 85% (GEM) versus those in the GS arm: 82% (S-1) and 85% (GEM). In the GA arm, 48% of patients had one or more dose reductions, and the corresponding rate in the GS arm was 33%. Eventually, NAC was completed in 44 patients (92%) in the GA arm and 38 patients (83%) in the GS arm. The RDI data for each arm are presented in Table 2.

**Table 2:** Comparison between GS and GA arms

	GS arm	GA arm	*P*-value
	*N*, ratio or mean ± SD		
*Outcomes of NAC treatment*			
BSA (m2)	1.55 ± 0.03	1.56 ± 0.03	0.654
Relative dose intensity of GEM (%)	84.8 ± 3.1	84.8 ± 3.0	0.961
Relative dose intensity of S-1 or nPTX (%)	82.3 ± 3.4	84.5 ± 3.4	0.655
Any grades of adverse events (*n*, %)**	42, 91.3%	45, 93.8%	0.651
G3/4 adverse events (*n*, %)**	36, 78.3%	35, 72.9%	0.547
Reduction rate of the tumor diameter (%)^§^	−8.6 ± 3.5	−15.8 ± 3.4	0.141
Reduction rate of CA19-9 (%)^§^	**−21.4 ± 8.0**	**−50.3 ± 8.0**	**0.012**
Reduction rate of CEA (%)^§^	116.2 ± 52.3	41.8 ± 52.3	0.317
Reduction rate of DUPAN-2 (%)^§^	−1.5 ± 11.3	−17.0 ± 12.6	0.363
Completion of NAC (*n*, %)	38, 82.6%	44, 91.7%	0.185
Resection rate (*n*, %)	33, 71.7%	41, 85.4%	0.103
*Surgical outcomes and adjuvant chemotherapy*			
PD/DP/TP	20/11/2	31/9/1	0.356
PV/SMV resection	10	13	0.547
Major arterial resection	1	0	0.304
Operation time, min	469 ± 27.7	486 ± 24.9	0.645
Blood loss, ml	662 ± 134.1	650 ± 120.3	0.946
Surgical morbidity (+)^§§^	8, 24.2%	8, 19.5%	0.624
POPF (+)^§§§^	6, 18.2%	4, 9.8%	0.293
Reoperation (+)	2, 6.1%	3, 7.3%	0.830
Surgical mortality	0, 0.0%	0, 0.0%	–
Adjuvant chemotherapy (+)	30, 90.9%	35, 85.4%	0.468
Adjuvant chemotherapy			
(S-1/GEM based)	27/3	31/4	0.853
Completion of adjuvant chemotherapy (+)	24, 72.7%	29, 70.7%	0.850
*Pathological findings and recurrence in patients with resection*			
R0/R1,2	30/3	38/3	0.782
UICC pT (0/1/2/3/4)*	0/18/14/1	1/24/15/1	0.821
UICC pN (0/1+2)*	11/22 (9+13)	23/18 (10+8)	0.051
Evans classification (I+IIa/IIb+III+IV)	19/14	28/13	0.342
Number of metastatic lymph nodes	**3.2 ± 0.5**	**1.7 ± 0.5**	**0.037**
Recurrence (−/+)	9/24	16/25	0.434
Initial recurrence type (local/metastasis/both)	4/17/3	7/12/6	0.264
Initial recurrence site (local/liver/lung/LN/peritoneal//multiple)	7/10/1/5/7//5	13/7/3/4/5//6	–
*Severe adverse events observed in each arm*			
**G3/4/5 adverse events**			
Hematological	31 (67)	36 (75)	0.415
Leukopenia	14 (30)	22 (46)	0.125
Neutropenia	25 (54)	34 (71)	0.098
Thrombocytopenia	5 (11)	8 (17)	0.415
Anemia	1 (2)	0 (0)	0.304
Non-hematological	12 (26)	9 (19)	0.393
Rash	2 (4)	1 (2)	0.532
AST/ALT increase	4 (9)	4 (8)	0.950
Hyperbilirubinemia	0 (0)	2 (4)	0.162
Febrile neutropenia	1 (2)	1 (2)	0.976
Creatinine increase	0 (0)	0 (0)	–
Anorexia	3 (7)	0 (0)	0.072
Constipation	0 (0)	1 (2)	0.325
Diarrhea	2 (4)	0 (0)	0.144
General fatigue	0 (0)	0 (0)	–
Stomatitis	1 (2)	0 (0)	0.304
Hair loss	0 (0)	0 (0)	–
Peripheral neuropathy	0 (0)	0 (0)	–
Others	3 (7)	4 (8)	0.738

Bold value indicates statistically significant differences

^*^* Data on adverse events were collected according to the CTCAE 4.0 classification.

^§^Reduction rates were calculated by dividing the value after NAC treatment by that before the start of NAC treatment.

^§§^Surgical morbidity data were collected according to Clavien–Dindo classification, and clinically relevant morbidity (grade IIIa or above) was included in ‘(+).’

^§§§^POPF data were collected according to the ISGPF (2016) classification, and clinically relevant POPF (grade B or above) was included in ‘(+).’

^*^ TNM classification was according to the 8th UICC classification.

^¶^ Data on adverse events were collected according to the CTCAE 4.0 classification.

*GS arm* gemcitabine + S-1 regimen arm, *GA arm* gemcitabine + nab-paclitaxel arm, *BSA* body surface area, *GEM* gemcitabine, *nPTX* nab-paclitaxel, *NAC* neoadjuvant chemotherapy, *PD* pancreatoduodenectomy, *DP* distal pancreatectomy, *TP* total pancreatectomy, *PV* portal vein, *SMV* superior mesenteric vein, *POPF* postoperative pancreatic fistula, *GEM-based* gemcitabine-based chemotherapy including monotherapy

### Surgery

Surgery was performed in 82 patients according to the preoperative images, and 8 patients did not undergo resection due to the detection of various intraoperative factors. Eventually, 74 patients received pancreatectomy with curative intent, and 68 (92%) of those procedures were R0 resections. Surgical morbidity and mortality rates were 19% and 0%, respectively, and there was no difference between arms. The details of the surgical outcomes of each arm are presented in Table 2.

### Subsequent Therapy

After resection, 85% of patients received AC in the GA arm (*N* = 35), and the corresponding rate in the GS arm was 91% (*N* = 30); 29 patients in the GA arm (71%) and 24 patients in the GS arm (73%) completed AC as planned by each corresponding physician, and there was no significant difference (*P* = 0.850, Table 2). Of these, 89% of patients in the GS arm were administered S-1, and 90% of patients in the GA arm were administered S-1. Otherwise, the remaining patients were administered GEM or GEM-based therapy after resection as AC (Table 2).

In total, 12 patients who did not undergo resection due to tumor progression in the GS arm received subsequent treatment; 8 of those received the GEM+nPTX regimen (67%), 3 of those received fluorouracil-based regimens, including the modified FOLFIRINOX regimen (25%), and 1 patient received the GEM-based regimen (8%). Six patients who did not undergo resection due to tumor progression in the GA arm received subsequent treatment; three received gemcitabine-based regimens, including GEM+nPTX or GEM monotherapy (50%), and three received fluorouracil-based regimens, including modified FOLFIRINOX (50%).

### Efficacy

#### Primary Endpoint: PFS

**Fig. 2:**
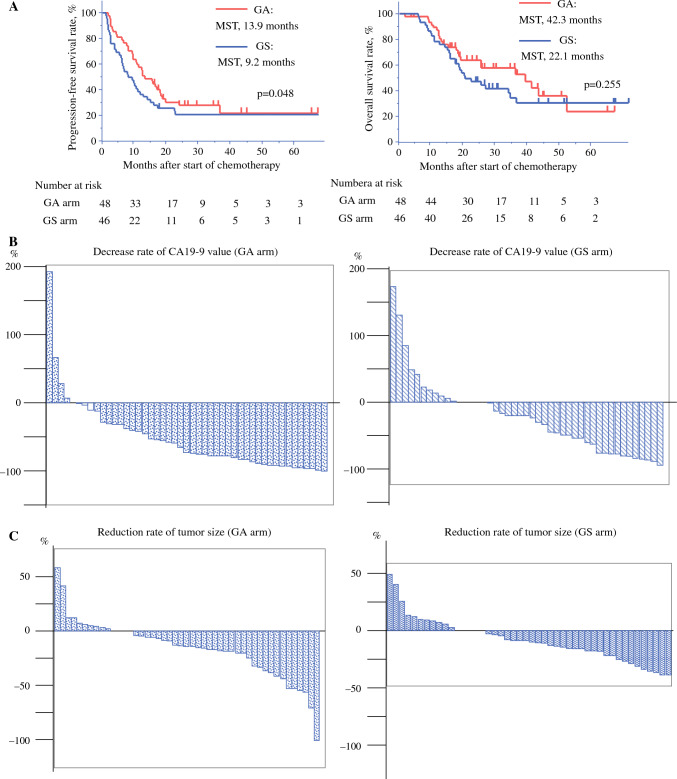
Responses and outcomes; Kaplan‒Meier survival curves of progression-free survival (PFS) and overall survival (OS) of patients divided into GA/GS arms (**A**), both PFS and OS were evaluated by intention-to-treat analysis, waterfall plot of the rate of decrease in tumor marker CA19-9 (**B**) and the tumor shrinkage rate (**C**) comparing after/before NAC treatment of each regimen, *MST* median survival time, *GA arm* gemcitabine + nab-paclitaxel arm, *GS arm* gemcitabine + S-1 regimen arm

After resection, 49 patients developed recurrence until the cutoff date. The median PFS in all patients was 12 (9–16) months (Supplementary Fig. 1). The median PFS of patients in the GA arm was 14 (10–20) months and that of patients in the GS arm was 9 (6–14) months. The rate of PFS at 2 years after allocation was better in the patients of GA arm but did not reach 10% increase (GA arm 31%, GS arm 26%). The GA arm showed a modest improvement in PFS compared with the GS arm in Kaplan‒Meier analysis (Fig. 2A, *P* = 0.048, Wilcoxon) and in Cox proportional hazards model (HR 0.71, 95% CI 0.45–1.12, refer to GS arm).

### Secondary Endpoints

The median OS in all patients was 29 (20–42) months (Supplementary Fig. 1). The median OS of patients in the GA arm was 42 (20–56) months and that of patients in the GS arm was 22 (17–37) months. The difference between arms was not statistically significant (Fig. 2A, *P* = 0.255, Wilcoxon) or in Cox proportional hazards model (HR 0.76, 95% CI 0.44–1.31, refer to GS arm).

The average reduction rate of the tumor marker CA19-9 showed a significant difference of −50.3% in the GA arm and −21.4% in the GS arm (*P* = 0.01, Fig. 2B). The average reduction rate of the tumor diameter was −15.8% in the GA arm and −8.6% in the GS arm (*P* = 0.14, Fig. 2C).

In the patients with resection, pathological findings showed a significant difference in the average number of lymph node metastases (GA/GS arm, 1.7 ± 0.5/3.2 ± 0.5, *P* = 0.04). The details of the responses are presented in Table 2.

The resection rate of each arm was 85% in the GA arm and 72% in the GS arm, and there was no statistically significant difference (*P* = 0.10, Table 2). AC following surgery was performed in 85% of patients in the GA arm and in 91% of patients in the GS arm, and the performance rate was not significantly different (*P* = 0.468, Table 2).

Of the 49 patients with recurrence, the initial recurrence types were local, distal metastasis, and both, and the numbers were 11 (15%), 29 (39%), and 9 (12%), respectively, and there was no significant difference in the rate of both arms (*P* = 0.264). The details of the initial recurrence site are presented in Table 2.

### Subgroup Analysis

**Fig. 3:**
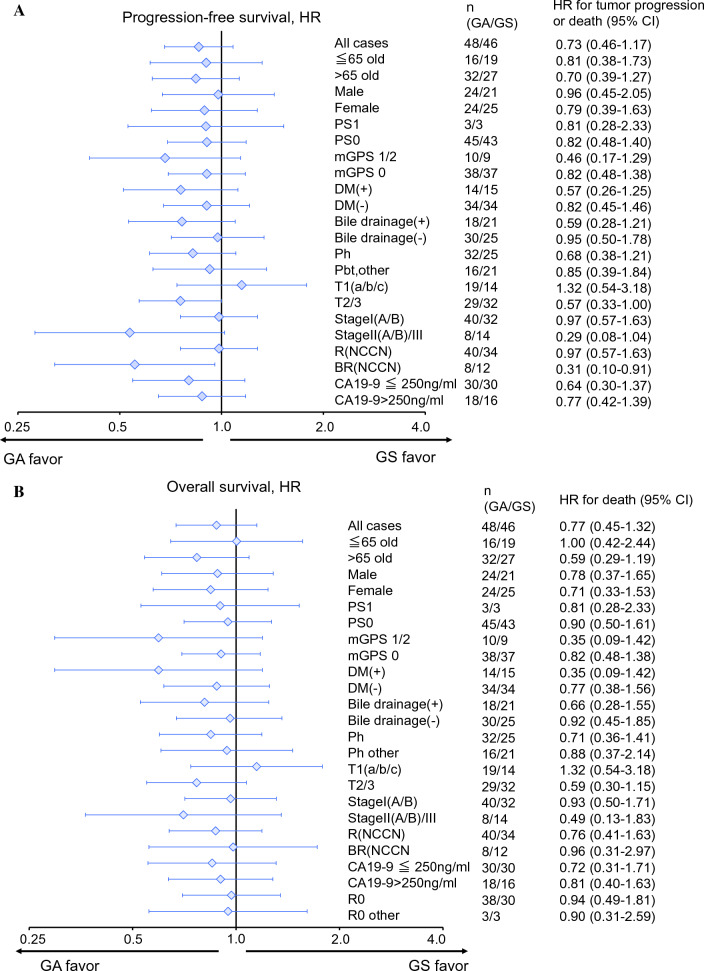
Forest plot subgroup analysis of PFS (**A**) and OS (**B**)

Subgroup analyses of PFS or OS are depicted in Figs. 3A and 3B. Generally, the HR in PFS did not differ between arms except T2/3 or BR-PDAC, in which the HR of the GA arm side was preferable.

### Safety

#### Adverse Events (AEs) of NAC

The details of AEs of NAC are listed in Table 2 and Supplementary Tables 1 and 2. Any grade of AEs was detected in 94% of patients in the GA arm and 91% of patients in the GS arm. Severe AEs were detected in 73% of patients in the GA arm and 78% of patients in the GS arm, and there was no difference between arms (*P* = 0.55). One patient in the GA arm died because of severe drug-induced interstitial pneumonia, and one patient in the GS arm discontinued treatment because of liver injury with severe fatty infiltration. The most frequent severe AEs in the GA arm versus the GS arm were neutropenia (71% versus 54%) and thrombocytopenia (17% versus 11%), whereas complaints of anorexia were more common in the GS arm (0% versus 7%).

## Discussion

This randomized, multicenter study showed that two cycles of GA regimen and two cycles of GS regimen were both feasible NAC regimens for patients with R/BR-PDAC. While the primary endpoint was not met, GA resulted in improved median PFS and other significant secondary endpoints.

Our reported clinical benefits with the GA/GS regimen as NAC treatment for localized PDAC are consistent with the results of other trials. Table 3 presents the results of major previous RCTs and our research.^2, 3, 11, 13, 17, 19, 26–31^ In the NEONAX and SWOG-1505 trials,^19, 30^ six cycles of GA regimen treatment were divided into NAC and AC and administered to patients with R-PDAC, referring to the APACT trial.^11^ Both clinical trials with the NAC-GA arm demonstrated modest clinical benefits (OS/PFS/resection rate, 24–26 months/12 months/70%). Those results were consistent with our results in the GA arm (OS/PFS/resection rate, 42 months/14 months/85%), supporting the finding that the GA regimen as NAC for localized PDAC is consistently feasible, with modest clinical benefits. Furthermore, in the PREP-02 trial, Unno et al. demonstrated a resection rate of 81% and a median OS of 37 (29–43) months in patients using the GS regimen as NAC for R/BR-PDAC.^17^ Since the median follow-up time of our study was still 22 months, and the median OS of 22 months in the GS arm is anticipated to be prolonged with a longer observation time, we assume that their results were also in line with our results [resection rate/OS, 72%/22 (17–37) months]. Although not statistically significant, GA arm exhibited higher rates of NAC completion and successful resection. This could potentially account for the observed differences in PFS. The underlying reason appeared to be the enhanced disease control capability. In GA arm, progression during chemotherapy was detected in 6 patients, whereas in GS arm, progression was observed in 12 cases.

**Table 3:** Results of major RCTs concerning perioperative treatment for R/BR PDAC and our results

Source	Patient		NAC or upfront surgery		Resection rate, %	AC		PFS*	OS
	R/BR	*N*	Regimen	Rate, %**		Regimen	Rate, %**	Median, months (95% CI)	
CONKO-001 (phase III)	R/BR^§^	179	Upfront	–	–	GEM	62%	13 (12–15)	23 (NA)
		175	Upfront	–	–	–	–	7 (6–8)	20 (NA)
JASPAC-01 (phase III)	R/BR^§^	187	Upfront	–	–	S-1	72%	23 (NA)	47 (38–64)
		190	Upfront	–	–	GEM	58%	11 (10–14)	26 (23–30)
ESPAC-4 (phase III)	R/BR^¶^	364	Upfront	–	–	GEM+Cape	54%	14 (12–17)	28 (24–32)
		366	Upfront	–	–	GEM	65%	13 (12–15)	26 (23–28)
APACT (phase III)	R/BR^¶^	432	Upfront	–	–	GEM+nPTX	66%	17 (NA)	42 (NA)
		434	Upfront	–	–	GEM	71%	14 (NA)	38 (NA)
PRODIGE24 (phase III)	R/BR^¶^	247	Upfront	–	–	mFOLFIRINOX	66%	21 (18–27)	54(22–NE)
		246	Upfront	–	–	GEM	79%	13 (12–15)	36 (20–81)
NEONAX (phase II)	R	59	GEM+nPTX	90%	70%	GEM+nPTX	64%	12 (9–15)	26 (20–30)
		59	Upfront	–	78%	GEM+nPTX	34%	6 (4–12)	17 (12–22)
PREOPANC (phase III)	R/BR	119	GEM+RT	89%	61%	GEM	62%	8 (NA)	16 (13–21)
		127	Upfront	–	72%	GEM	53%	8 (NA)	14 (13–18)
PREP02 (phase III)	R/BR	182	GEM+S-1	ND	81%	S-1	ND	ND	37 (29–43)
		180	Upfront	–	73%	S-1	ND	ND	27 (21-31)
SWOG 1505 (phase II)	R	47	GEM+nPTX	85%	70%	GEM+nPTX	40%	NA	24 (18–32)
		55	mFOLFIRINOX	84%	73%	mFOLFIRINOX	49%	NA	23 (18–45)
NORPACT-1 (phase II)	R	77	FOLFIRINOX	NE	82%	GEM+Cape/ GEM/ mFOLFIRINOX (oncologist’s discretion)	69%	12 (9–16)	25 (17–35)
		63	Upfront	–	89%		63%	16 (11–21)	39 (28–not reached)
Our study (phase II)	R/BR	48	GEM+nPTX	92%	85%	Mainly S-1	71%	14 (10–20)	42 (20–56)
		46	GEM+S-1	83%	72%	Mainly S-1	73%	9 (6–14)	22 (17–37)

^*^PFS was diverted from the disease-free survival described in the reports of upfront surgery + adjuvant chemotherapy.

^**^The completion rate of NAC/AC is depicted.

^§^The details of the BR-PDAC patients enrolled in the studies were unclear. There was a possibility that the studies included patients with initially UR-LA PDAC.

^¶^Several patients with UR-PDAC were included in those studies without intent.

*NAC* neoadjuvant chemotherapy, *AC* adjuvant chemotherapy, *PFS* progression-free survival, *OS* overall survival, *R* resectable, *BR* borderline resectable, *CI* confidence interval, *GEM* gemcitabine, *NA* not assessed, *Cape* capecitabine, *GEM+nPTX* gemcitabine+ nab-paclitaxel, *mFOLFIRINOX* modified FOLFIRINOX, *NE* not evaluated, *ND* not described

The principle of perioperative chemotherapy for PDAC, which seems to localize, is to kill the tumor seeds that spread and hide in the patient’s body and to reduce recurrence after radical surgery. For this purpose, it is desirable to use a potent regimen. However, there is a concern that NAC regimens may negatively affect surgery in the preoperative setting, whereas the AC regimen may cause issues with treatment tolerance in the postoperative setting. From this aspect, the resection rate and completion rate of AC are crucial aspects in perioperative chemotherapy studies, and NAC treatment of both arms did not compromise these outcomes, which is endorsed by similar results from previous reports (Table 3).^2, 3, 11, 13, 17, 19, 26–30^ Moreover, the event rates of postoperative complications were not impaired in patients receiving either regimen (GA/GS, 20%/24%) compared with those rates reported in the patients treated by upfront surgery (17–65%).^14, 16^ Thus, this trial at least indicated that both NAC regimens were safely performed without impairing perioperative outcomes.

Several factors with patients in the GA group showed various preferable findings in terms of secondary endpoints, moreover, the GA regimen indicated significant superiority to the GS regimen in PFS by Kaplan–Meier analysis, and it might be expected that NAC with the GA regimen would be a better regimen for localized PDAC in future studies with more patients. Since we intended to explore the optimal NAC regimen for patients with early localized PDAC (i.e., R-PDAC), the next trial should be examined with patients with pure R-PDAC.

We expected that better NAC treatment would result in at least a 10% increase in the rate of PFS at 2 years, but the primary endpoint was not reached. Referring to the result of PREOPANC-1 trials (preoperative chemo-radiation treatment (CRT) arm, *n* = 119, versus immediate surgery arm, *n* = 127), NAC treatment demonstrated a significant improvement in OS compared with upfront surgery for R/BR-PDAC. However, the difference of PFS at 2 years appeared to be only 9%.^29^ Therefore, it was imperative to set more stringent difference, and the calculated sample size should have been increased. Consequently, the design of the next phase III trial should include a larger number of patients with pure R-PDAC.

While the primary endpoint was not achieved, GA led to improved median PFS and other important secondary endpoints. Although further research is needed, GA should be considered a standard NAC regimen for R/BR-PDAC.

### Supplementary Information

Below is the link to the electronic supplementary material.

Supplementary file1 (PDF 132 KB)

## Data Availability

The data that support the findings of this study are available from the corresponding author, S.K., upon reasonable request. Individual participant data will not be available. Individual participant data that underlie the results reported in this article will be shared after deidentification. Data will be available beginning 9 months and ending 36 months following article publication.
