# Supplementary material for: Results of a Randomized Clinical Study of Gemcitabine Plus Nab-Paclitaxel Versus Gemcitabine Plus S-1 as Neoadjuvant Chemotherapy for Resectable and Borderline Resectable Pancreatic Ductal Adenocarcinoma (RCT, CSGO-HBP-015)
Source: Ann Surg Oncol. 2024 Mar 28;31(7):4621–33. doi: 10.1245/s10434-024-15199-8 (PMC11164807; doi:10.1245/s10434-024-15199-8)
Supplement: Supplementary file 1 — Supplementary file1 (PDF 132 KB) [file 10434_2024_15199_MOESM1_ESM.pdf]

Supplementary Table1 The details of adverse events with GA regimen

GA arm	Grade (CTCAE version 4.0)						
	0	1	2	3	4	5	G3–5 (%)
Hematological							36 (75)
Leukopenia	12	2	12	20	2	0	22 (46)
Neutropenia	7	2	5	19	15	0	34 (71)
Thrombocytopenia	23	14	3	8	0	0	8 (17)
Anemia	12	22	14	0	0	0	0 (0)
Non hematological							9 (19)
Rash	45	2	0	1	0	0	1 (2)
AST/ALT increase	31	11	2	4	0	0	4 (8)
Hyperbilirubinemia	38	6	2	2	0	0	2 (4)
Febrile neutropenia	47	-	-	0	1	0	1 (2)
Creatinine increase	48	0	0	0	0	0	0 (0)
Anorexia	34	9	5	0	0	0	0 (0)
Constipation	31	13	3	1	0	0	1 (2)
Diarrhea	43	4	1	0	0	0	0 (0)
General fatigue	31	14	3	0	0	0	0 (0)
Stomatitis	44	3	1	0	0	0	0 (0)
Hair loss	21	10	17	0	0	0	0 (0)
Peripheral neuropathy	33	15	0	0	0	0	0 (0)
Others	44	1	0	2	1	1	4 (8)

Supplementary Table 2 The details of adverse events with GS regimen

GS arm	Grade (CTCAE version 4.0)						
	0	1	2	3	4	5	G3–5 (%)
Hematological							31 (67)
Leukopenia	15	4	13	13	1	0	14 (30)
Neutropenia	10	0	11	18	7	0	25 (54)
Thrombocytopenia	29	9	3	3	2	0	5 (11)
Anemia	15	24	6	1	0	0	1 (2)
Non hematological							12 (26)
Rash	40	2	2	2	0	0	2 (4)
AST/ALT increase	39	1	2	4	0	0	4 (9)
Hyperbilirubinemia	34	9	3	0	0	0	0 (0)
Febrile neutropenia	45	-	-	1	0	0	1 (2)
Creatinine increase	45	1	0	0	0	0	0 (0)
Anorexia	29	12	2	3	0	0	3 (7)
Constipation	34	9	3	0	0	0	0 (0)
Diarrhea	43	1	0	2	0	0	2 (4)
General fatigue	28	15	3	0	0	0	0 (0)
Stomatitis	36	7	2	1	0	0	1 (2)
Hair loss	41	5	0	0	0	0	0 (0)
Peripheral neuropathy	45	1	0	0	0	0	0 (0)
Others	42	1	0	3	0	0	3 (7)

Supplementary Figure 1

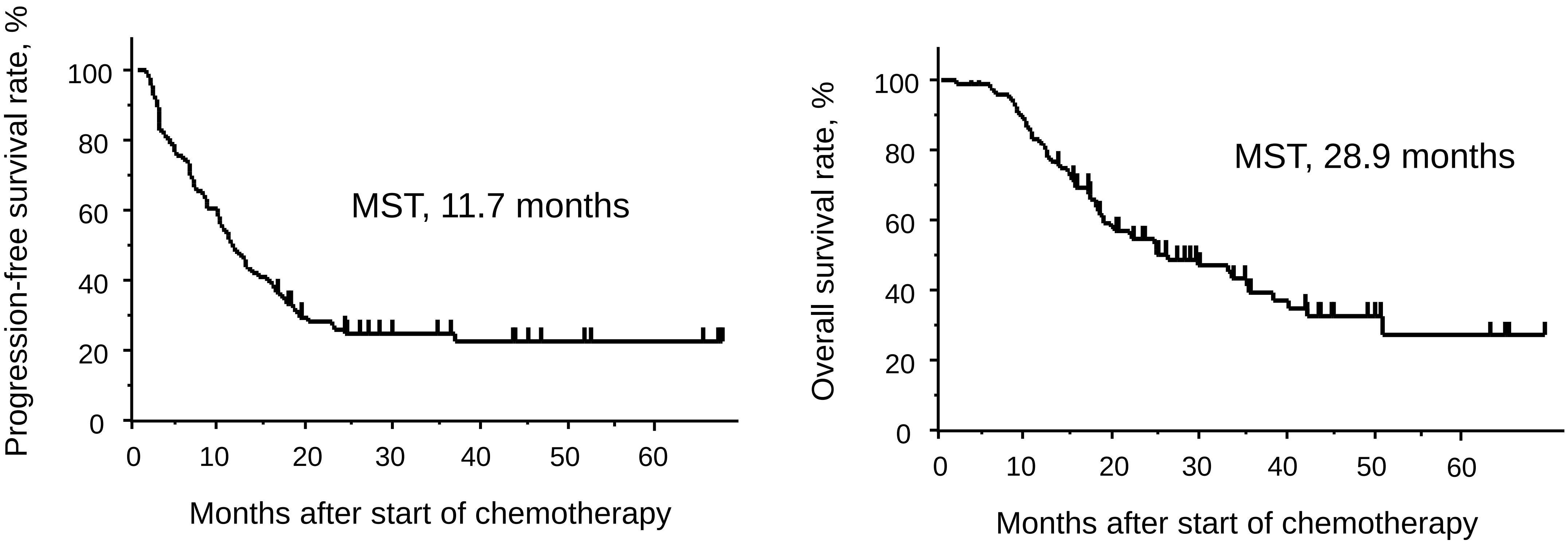

Number at risk						
94	55	28	15	10	6	4

Number at risk						
94	84	56	32	19	11	5

Responses and outcomes. Kaplan–Meier survival curves of progression-free survival (PFS) and overall survival (OS) of all patients
